# Milk: Is Its Sterilizing Necessary?

**Published:** 1892-08

**Authors:** 


MILK: IS ITS STERILIZING NECESSARY?—Dr. Freudenreich, after a series of experiments on the action of raw milk on bacteria, has come to the conclusion that it possesses remarkable germicidal properties. He claims that the bacillus of cholera in fresh cow's milk dies in an hour; the bacillus of typhoid fever in twenty-four hours, while other germs die at the end of varying periods. He further found that milk exposed to a temperature of 131 degs. F. loses this germicidal property, as also milk that is four or five days old.

These experiments will set the physicians to thinking very seriously on the advisability of sterilizing milk for infants' food, or for food of adults. We were just congratulating ourselves on the fact that a means of preventing the introduction of disease into the human body through milk had been discovered in sterilization. According to Dr. Freudenreich, one might conclude, at first thought, that we were mistaken in our expectations and confidence, and that raw milk is, after all, preferable for human consumption.—*Bacteriological World.*

